# Cultivating death literacy and compassionate communities via ‘The Omega course’: A mixed methods exploration

**DOI:** 10.1177/26323524261481869

**Published:** 2026-09-17

**Authors:** Suzannah Laws, Chantal Meystre, John MacArtney

**Affiliations:** 1 Warwick Medical School, 2707 University of Warwick, Gibbet Hill Campus, Coventry, UK; 2 The Omega Course Kenilworth, Warwickshire, UK

**Keywords:** compassionate communities, death education, death literacy, mixed methods, palliative care

## Abstract

**Background:**

The public health approach to palliative care argues developing death literacy and compassionate communities are key in meeting the increasing demand for palliative care. One potential means of building such skills is The Omega Course, a group educational intervention for healthy community members run across five sessions.

**Objectives:**

To explore the impact of The Omega Course on participant death literacy, and to explore whether the course promotes compassionate community development. A retrospective pre–post observational study design with quantitative analysis and embedded complementary qualitative analysis was used.

**Methods:**

Mixed-methods secondary analysis of 179 pre- and post-course questionnaires consisting of Likert scales and open-text responses collected between 2016 and 2024. Responses were analysed using statistical and conventional content analysis. Quantitative and qualitative integration was supported by a Stakeholder Engagement Meeting.

**Results:**

Paired t-tests (n=150) revealed statistically significant increases in comfort and preparedness for dying post-course across all six questions (all ps<0.001), suggesting the course successfully cultivates comfort and preparedness for death, as a proxy for death literacy. Content analysis identified that social contact (e.g. meeting other participants) and communication skill-building (e.g. roleplaying) were perceived as the best, yet simultaneously most challenging, aspects of the course.

**Discussion:**

Integration of findings suggests the shared challenge of social experiences may be what makes the course so successful in building death literacy and this can contribute to developing compassionate communities. The study was limited by participant homogeneity (predominantly older, female, and affluent). Structured educational interventions with a social element, such as The Omega Course, represent a potential step towards community preparedness for dying.

## Plain language summary

**Why did we do this study?** The number of people requiring medical and social care as they are dying is increasing. Some researchers suggest connections within our communities can help meet this need. For example, members of the community (e.g. friends, neighbours) could provide emotional support (e.g. visiting, talking), and/or practical support (e.g. cooking, helping with medications) to those who are dying. We wanted to know whether 'The Omega Course', a five-week educational course about dying and death, could help people feel more prepared to provide such support within their communities. **What did we do?** Between 2016 and 2024 179 Omega Course attendees completed questionnaires before and after the course indicating how prepared and comfortable they felt around death. They were also invited to write down the best and most challenging parts of the course. **What did we find out?** After completing the course, attendees felt more comfortable around death, and felt more prepared to provide support to those who are dying in their communities. The best and most challenging parts of the course were the social aspects. For example, many participants felt meeting and sharing stories with other attendees was the best part of the course. Simultaneously, many participants found ‘opening up’ to a group and navigating group discussions the most difficult part of the course. **Why are these results useful?** Our results suggest The Omega Course helps attendees feel more prepared to support people in their community who are dying, and that creating social connections and experiencing challenging social situations during the course was key to this success. Therefore, our results suggest we may be able to use educational courses which involve social interaction and connection, such as The Omega Course, to help support the increasing number of people needing care as they die.

## Introduction

It is estimated that by 2040 annual deaths in England and Wales will increase by at least 25%, representing almost 130,000 extra deaths per year.^1^ A large proportion of these deaths are predicted to involve serious and chronic suffering due to diseases such as cancer and dementia^2^ and are expected to increase the need for palliative and supportive care.^1^

### The importance of being ‘death literate’

Death literacy is the knowledge, skills, experiences, and actions allowing one to access, understand and navigate palliative support.^3^ The concept of death literacy stems from the Public Health Approach to palliative care, which itself was developed in response to the World Health Organisation’s redefinition of health as not merely the absence of illness, but as a holistic concept encompassing physical, mental, and social well-being.^4^ The Public Health Approach moves beyond a medical focus upon dying to emphasise dying as an important part of life that requires a coordinated response by all sectors of society.^5^ Therefore, to provide such support and improve the dying experience for the increasing number of people requiring end-of-life care, it has been argued that widespread death literacy is needed,^2,3^ as greater death literacy should enable individuals to improve the quality of life of others (and themselves) who are dying. By being death literate it is hoped individuals will be able to provide practical support, such as gaining access to palliative care services, assisting with organisation and legalities, and providing personal care, as well as social and emotional support, such as empathetic listening.^3^

As it is, more than two-thirds of UK adults have never told anyone their end-of-life care preferences, and similarly do not know the preferences of their family members.^6^ Additionally, only 14% of UK adults have formally documented their end-of-life wishes, and most of these are made in the final stages of life.^7^ Furthermore, although one in three patients acutely admitted to hospital are in their last year of life, it was found that only 4% of those that died had recorded or discussed their preferences preadmission.^8^ Such statistics are unfortunate, given that discussing death (i.e. cultivating death literacy), and formal documentation of end-of-life preferences are associated with more positive, personalised experiences whilst dying, and a familial perception of ‘dying well’.^9–11^ It is in this context that collaborative community-based approaches to death literacy education for the general public have developed.

### Building death literacy through compassionate communities

It is hypothesised that death literacy is most frequently acquired through experiential learning^3^: those with experience related to death and dying, e.g. people who have cared for people who are dying in a voluntary, paid, or personal capacity, score higher on measures of death literacy.^12,13^ However, many individuals do not have such exposure.^13^ Fortunately, there is appetite for death literacy development, with 72% of UK adults wanting to talk about death more openly.^14^

A facet of the Public Health Approach to developing death literacy is compassionate communities. These are understood as networks of friends, family, neighbours, and even strangers, who support those who are dying (and their carers) by providing social connection and practical support.^15^ Ideally, compassionate communities become self-sustaining with a broad remit, meeting all the needs of those who are dying through collaboration and skill sharing, with professional services acting as backup.^16^ It is proposed that compassionate community formation enables greater proliferation of death literacy, and simultaneously, greater death literacy enables compassionate community formation.^3^ Indeed, those who have experienced serious illness and/or death within their family are more likely to show greater willingness to support to other community members at the end of their lives.^17^

Various initiatives attempting to promote death literate compassionate communities exist e.g. death cafés, befriending schemes, and training courses.^18,19^ The most thoroughly documented of these is ‘Last Aid’ a structured four-module death education course which first ran in Norway in 2014 and has since been attended by thousands globally.^18,20^ The course has been extensively documented as positively received and feasible amongst adults and young people.^18,21^ However, the impact of the course on participant attitudes and behaviours has been less deeply explored. One content analysis of interviews with five Last Aid attendees found the course reduced fear around death, and that the success of the course was partly built upon sharing mutual experiences with other course attendees.^22^ Another interview study with 11 Last Aid attendees and facilitators found that the course improved participant’s practical knowledge of death and prompted them to think about their own end-of-life wishes, and that connectedness within the group was important in these learning experiences.^23^

The Omega Course is a UK-based community death educational intervention which is similar to Last Aid in that it seeks to cultivate death literacy and compassionate communities. Previous analysis of 23 Omega Course participants’ retrospective ratings found fears of death decreased following the course, and comfort and preparedness increased. In addition, thematic analysis of qualitative feedback revealed participants gained confidence in talking about death and felt they gained communication skills through group activities.^24^ These findings suggested the course may be effective in building death literacy and providing opportunity for compassionate community development via group social connection.

Death education courses therefore appear to be feasible interventions that provide positive experiences which can improve participant’s subjective understanding and fear around death.^18,21,22^ In addition, these findings indicate connectedness with other course attendees may be an important factor in achieving these improvements,^22,23^ suggesting such death education courses may also provide opportunity for compassionate community development. However, these studies’ small sample sizes have limited statistical quantitative evaluation of the impact of death education courses on attitudes towards death. Similarly, integrated qualitative exploration of compassionate community creation within such courses remains unexplored.

### Aim

The aim of the study was to identify if The Omega Course could improve participant comfort and preparedness around death as a proxy for death literacy, explore what participants reported were the key features of their experience, and consider if such initiatives can help support the development of a compassionate community.

### Hypotheses

#### Experimental

1. Self-reported comfort and preparedness around dying will significantly increase pre- to post-course.
2. Participants will highlight social connections as key parts of their experience, providing evidence for compassionate community creation.

#### Null

1. Self-reported comfort and preparedness around dying will not significantly change pre- to post-course.
2. Participants will not highlight social connections as key parts of their experience.

## Methods

### Design

This study is an explanatory sequential mixed-method study involving a secondary analysis of data collected by the second author between 2016-24. A retrospective pre–post observational study with embedded, complementary qualitative analysis is used. A post-positivist epistemological approach was used.

In accordance with Enhancing the QuAlity and Transparency Of Health Research (EQUATOR) reporting guidelines,^25^ the quantitative components of this study are reported in accordance with Strengthening the Reporting of Observational Studies in Epidemiology (STROBE) guidelines^26^ and the qualitative components in accordance with the Standards for Reporting Qualitative Research (SRQR).^27^ In addition, the mixed method integration was reported in accordance with Good Reporting of a Mixed-Methods Study (GRAMMS)^28^ (See Supplementary Material).

### Study setting: The Omega course

Founded in 2016, this UK-based intervention aims to educate and prepare participants for death and dying. It involves group discussions, presentations, and role-play scenarios, and typically runs over four weekly evening sessions (2-3 hours) and one extended session (6-7 hours) (for curriculum, See Supplementary Material).^29^

The course is primarily facilitated by its founder, a retired Palliative Care Consultant Doctor and co-author, and is co-facilitated by volunteer(s) from non-healthcare backgrounds e.g. teaching, counselling, and probation. Unlike many death literacy interventions,^19^ the course is independent of healthcare services and is primarily aimed at those who are currently healthy, without terminal diagnoses. It is open to any adult deeming themself to have capacity.^29^

Courses are advertised via social media, print, and word-of-mouth. Each course accommodates up to 18 attendees and takes place in a local venue e.g. cafes, church halls. One iteration of the course was run online due to Covid-19 social distancing restrictions. Whilst the first two courses in 2016 cost £65 per attendee, all subsequent courses were free and funded by local government and charitable grants, and independent donations.^29^

### Data collection

All course attendees to date were invited to complete pre-course (start of first session) and post-course (end of final session, before final plenary) questionnaires. For in-person courses, questionnaires were completed on paper in the same room as the course was held with other participants and facilitators present. For the online course, questionnaires were completed online via Google Forms software.

Questionnaires recorded date, location, self-described gender, and age. Paper questionnaires recorded exact age whereas online questionnaires recorded age range. Questionnaires contained six questions asking participants to rate their comfort and preparedness for death using a Likert scale between 1 (not comfortable at all) and 10 (totally comfortable). Post-course questionnaires also asked attendees to share the best and most challenging aspects of the course as free text responses (see Supplementary Material).

No personal data which could link participants to their responses (e.g. their name or date of birth) was retained, hence the data falls outside the UK General Data Protection Regulation (GDPR) requirements.^30^ Therefore, no explicit written consent from each participant was sought. However, in the interests of transparency, we note that each page of the questionnaire contained the following statement “We take your privacy very seriously. Please see our displayed privacy notice. We will use your data only to contact you with details of courses, events and occasional evaluations which may be used for research”.

The responses were de-identified and digitised by the second author. In October 2024, the anonymous data was transferred digitally to the first and third authors for secondary analysis.

### Analysis

Quantitative analysis was conducted using IBM SPSS version 29.0.2.0.^31^ After making parametric assumptions (see Supplementary Material 6), pre- and post-course scores were compared using two-tailed paired t-tests and Cohen’s d calculated. For each t-test, pairs containing missing data were excluded. In the interests of transparency and robustness, the equivalent non-parametric tests (Wilcoxon Signed Rank) were conducted (see Supplementary Material 6). As this was a retrospective secondary analysis, no a priori sample size calculation or power analysis was performed.

Qualitative analysis was conducted using conventional content analysis as described by Hsieh & Shannon^32^ using Microsoft Excel. Content analysis was completed by the first author, supervised by the third author (qualitative research expert). To ensure the codes were developed independently the second author was not involved in the preliminary analysis.

Responses from two questions were analysed: “Q7. What has been the best part of the course?” and “Q8. What has been the most challenging part of the course?” Data from each question were analysed separately. Initially for each question, all responses were read to gain an overview. Next, each response was divided into units of data. Units of data were defined as continuous strings of words capturing one idea about the course (see Figure 1, Columns 1 & 2).

**Figure 1.**
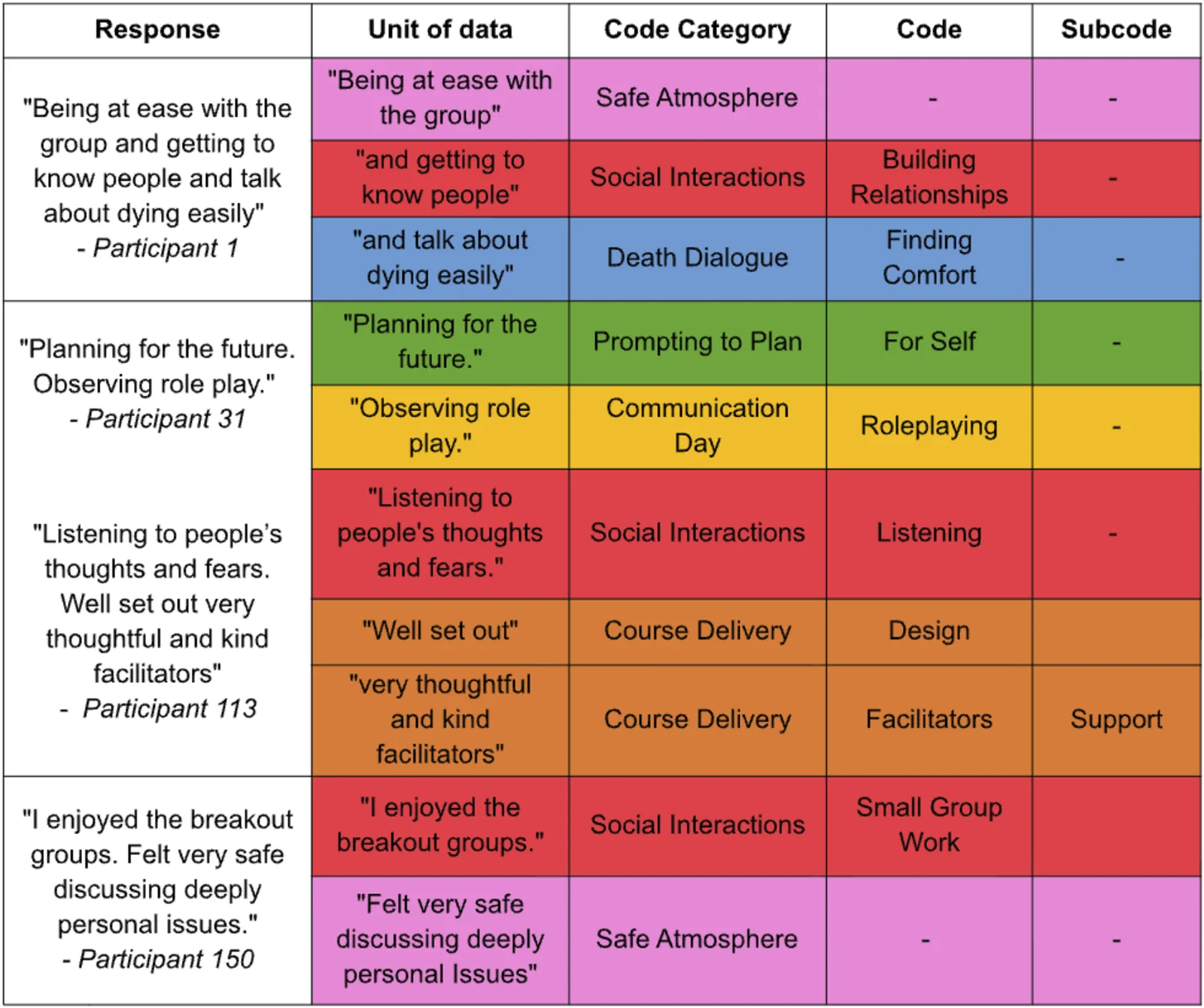
Table showing examples of how four participants’ qualitative responses to question 7 (What has been the best part of the course?) were divided into units of data and assigned to code categories, codes and subcodes. (-) indicates this data unit was not assigned a further code and/or subcode. Each colour corresponds to the named code category.

Open, inductive coding was used i.e. codes were not preconceived or based on previous literature. Each unit of data was assigned a code category (see Figure 1, Column 3). Further open, inductive coding within categories was used to organise units into codes, and where required, subcodes (see Figure 1 Columns 4 & 5).

All data was coded. There was no overlap between codes i.e. each unit of data was assigned one code only. Coding was iterative and revisited many times during collaboration between the first and third authors.

### Stakeholder engagement meeting

Following preliminary analysis, findings were presented by the first and third authors to the course founder (and second author) in a stakeholder engagement meeting. The course founder was invited to give feedback and provide context to the preliminary qualitative findings. The first and third authors used reflections, interpretations and feedback from this meeting to further refine data codes. However, the course founder was not involved in this refinement process and did not have any further input into the final results.

### Trustworthiness

Lincoln and Guba’s trustworthiness criteria^33^ were applied to enhance the qualitative and mixed method analyses.

#### Credibility

As previously described, there was prolonged and iterative engagement with the qualitative data by the first author over several months. Furthermore, consistent formal supervision and debriefing meetings were held between the first and third authors to enhance reflections upon and understanding of the data.

While member checks with participants were not possible due to the anonymity of the data (see Data Collection), the Stakeholder Engagement meeting was employed as previously described to allow the first and third authors to solicit reactions, discussions and clarifications with the second author, allowing data codes and subcodes to be further refined and reconstructed where required.

Triangulation of the qualitative data with qualitative data was also used as part of the mixed methods analysis to enhance credibility.

#### Transferability

All methodology employed in the study has been described in detail. In addition, example participant responses (see Results), descriptive figures (see Results), and a detailed code book (see Supplementary Material 7) have been included to enhance transferability of the content analysis process.

#### Dependability and confirmability

A pre-data collection protocol was not developed for this study. However, a clear audit trail was established: between 2016-24 the second author collected the questionnaire responses during each course. In October 2024, the anonymised data file was transferred digitally to the first and third authors for secondary analysis. The first and third authors carried out analysis as previously described. All data and materials are available on request via the Corresponding Author.

In addition, prior to analysis, the study’s conceptual basis and methodology was reviewed and critiqued by a qualitative and quantitative research specialist external to the project via a formal feedback process as part of the first author’s degree programme requirements.

### Funding and ethics

This study received no funding. Course delegated ethical approval was received from the University of Warwick Biomedical and Scientific Research Ethics Committee (reference: BSREC-CDA-SSC2-11).

## Results

### Descriptive results

179 pre-course and 155 post-course questionnaires were returned. Of all 184 course attendees in the sample, 100% completed at least one questionnaire.

Two respondents who were completing the course for a second time were excluded at the point of data collection to minimise response bias associated with repeated exposure to course content, which may have influenced their experience differently from first-time participants. No further exclusion criteria were applied.

The remaining 182 participants represent 17 courses occurring between 2016-24, 16 in-person (14 across four weeks, two across weekends) and one online (across four weeks). The in-person courses took place across several towns in the UK. Courses ranged from three to 18 participants.

131 participants were female and 51 male. For in-person courses, participant age ranged 22-93 years, with a median of 65 and mode of 68. For the online course, age range ranged 35-39 to 70-74, with a median and mode of 70-74.

#### Quantitative analysis

9.55% of data was missing (see Figure 2, Column 2). This data was missing due to participants electing not to complete all questionnaire items for unknown reasons, i.e. Missing Not at Random (MNAR).^34^

**Figure 2.**
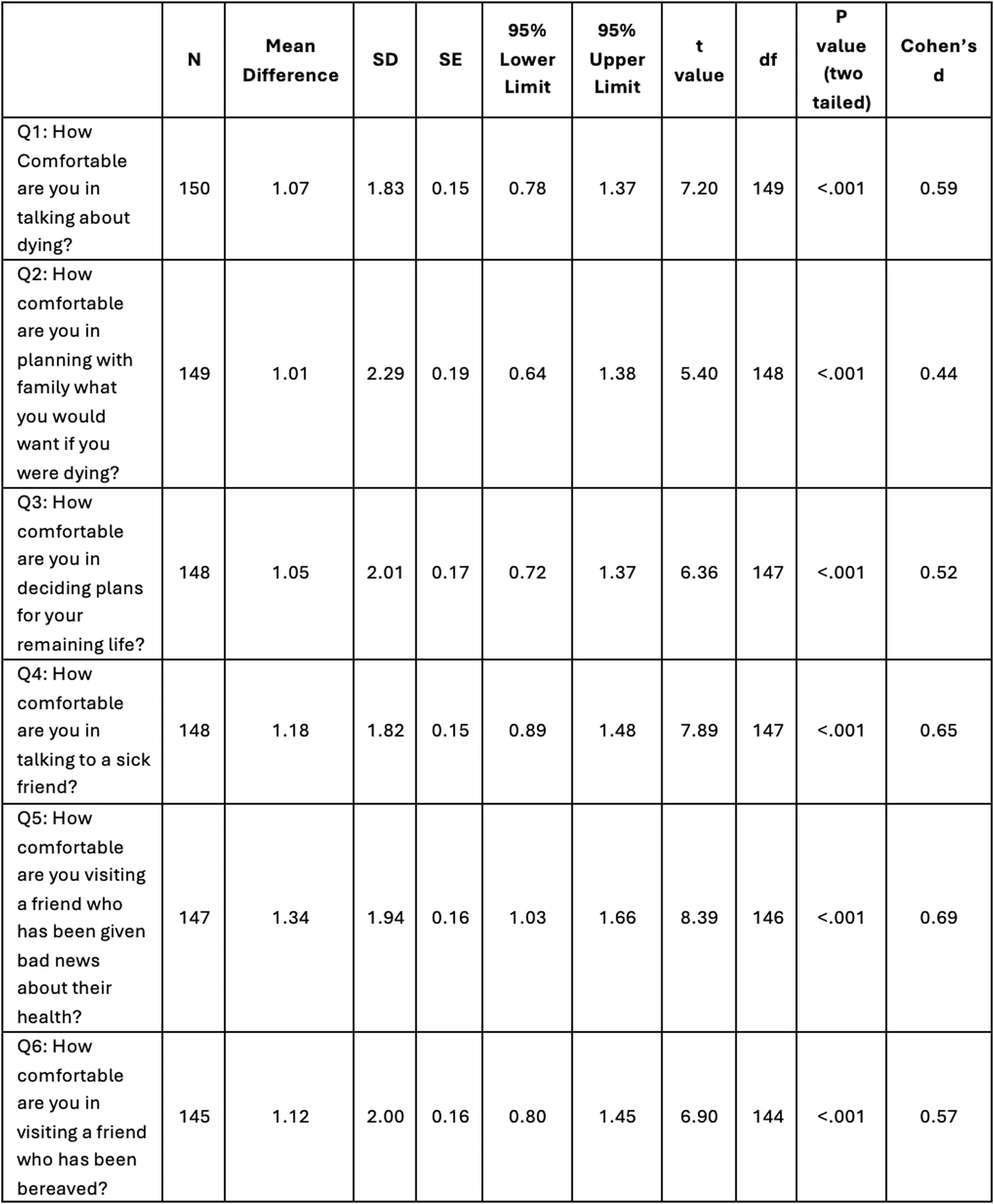
Table showing paired samples t-test results comparing pre- and post-course participant survey scores for questions 1-6. N = sample size. SD = standard deviation. SE = standard error of the mean. df = degrees of freedom.

Q1-6 scores significantly increased from pre-to post-course (all ps<0.001, see Figure 2, Column 10). In the interests of transparency and robustness, equivalent non-parametric tests (Wilcoxon Signed Rank) were conducted and revealed Q1-6 scores significantly increased from pre-to post-course (all ps<0.001, Supplementary Material 6).

Effect sizes (Cohen’s d) for Q1-6 ranged from 0.44 (Q2) to 0.69 (Q5). All effect sizes were small (>0.2) or medium (>0.5).^35^

### Qualitative analysis

Conventional content analysis of the two qualitative questions (“What has been the best part of the course?” and “What has been the most challenging part of the course?”) is presented below. Firstly, the responses to each individual question are described, and then the responses to both questions are synthesised.

#### What has been the best part of the course?

154 of 155 post-course questionnaires returned included a response to Question 7, one participant did not respond for unknown reasons.

329 units were coded. Units per response ranged 1-9 with an average of two. Nine code categories and 20 further codes were derived, of which two were further divided into sub-codes (see Figures 3 and 4 for all codes and totals, Supplementary Material for code book).

**Figure 3.**
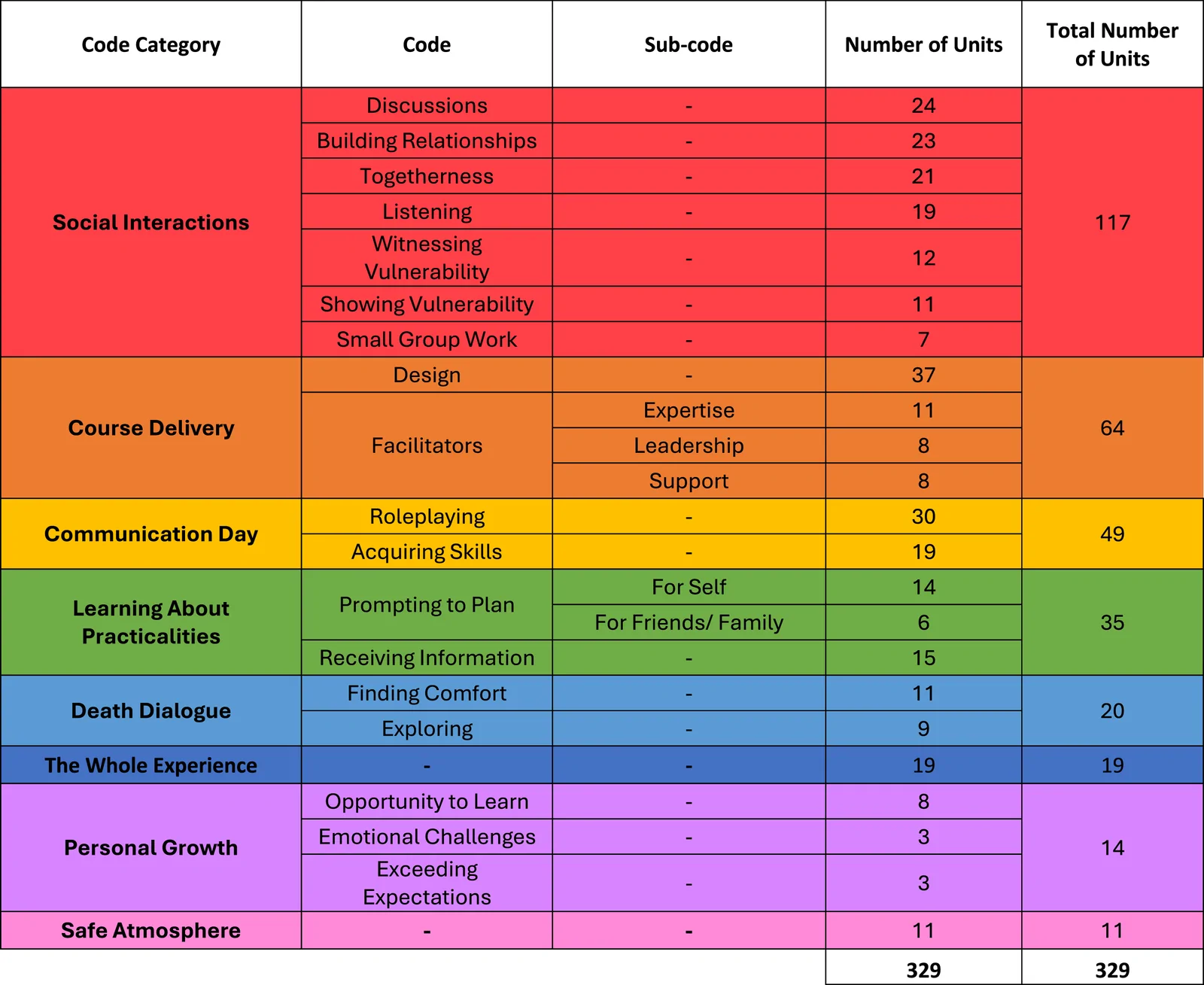
Table showing content analysis of responses to question 7 (what has been the best part of the course?) as number of data units for each code category, code and sub-code. (-) indicates a code and/or subcode was not required to further divide and describe the data. Each colour corresponds to the named code category.

**Figure 4.**
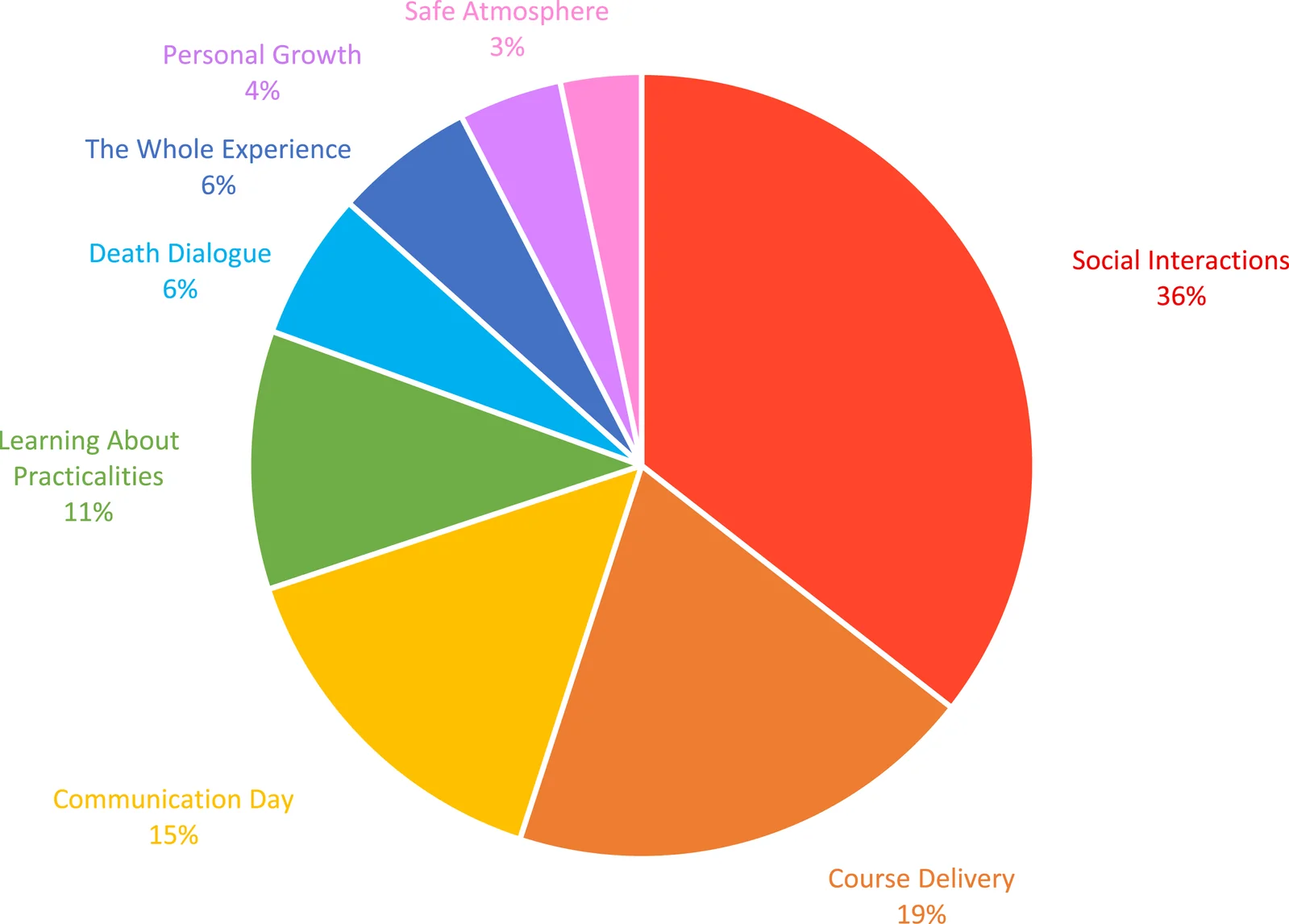
Pie chart showing content analysis of responses to question 7 (what has been the best part of the course?) by proportion (%) of data units assigned to each code category. Each colour corresponds to the named code category.

*Social Interactions* with other participants was the most prevalent code category, representing 117 units (36% of total). Participants commonly highlighted interactions such as *Discussions*, *Building Relationships*, feelings of *Togetherness*, and *Listening*.

“The shared conversations” – Participant 89 (*Social Interactions*, *Discussions)*

“Support offered from other members of the course” – Participant 39 (*Social Interactions, Togetherness)*

“Hearing real stories” *– Participant 10 (Social Interactions, Listening)*

*Course Delivery* was the second most prevalent category*,* representing 64 units (19% of total). 58% of these focused on course *Design,* and 42% on the *Facilitators* (describing their *Support, Expertise* and *Leadership*).

“It has been an excellent mix of practical, emotional, theoretical, role play etc.” – Participant 122 *(Course Delivery, Design)*

“Feeling so at ease with the facilitators” – Participant 47 *(Course Delivery, Facilitators, Support)*

The third most prevalent category was mention of the *Communication Day,* representing 49 units (15% of total). 61% of these highlighted *Roleplaying*, the remaining highlighted *Acquiring Skills:*

“The role play – v. powerful!” *–* Participant 135 *(Communication Day, Roleplaying)*

“Learning about options/ways to approach difficult situations with people” – Participant 21 *(Communication Day, Acquiring Skills)*

The fourth most prevalent category was *Learning About Practicalitie*s, representing 35 units (11% of total). 57% of these highlighted *Prompting to Plan*, more commonly *For Self* than *For Friends/Family*, and the remaining 43% highlighted *Receiving Information.* The remaining code categories were *Creating* a *Death Dialogue* (20 units, 6% of total), *The Whole Experience* (19 units, 6% of total), *Personal Growth* (14 units, 4% of total), and *Safe Atmosphere* (11 units, 3% of total).

#### What has been the most challenging part of the course?

149 of 155 post-course questionnaires returned included a response to Question 8, six participants did not respond for unknown reasons.

189 units were coded. Units per response ranged 1-3 with an average of one. Six code categories and 14 codes were derived (see Figures 5 and 6 for all codes and totals,see Supplementary Material for code book).

**Figure 5.**
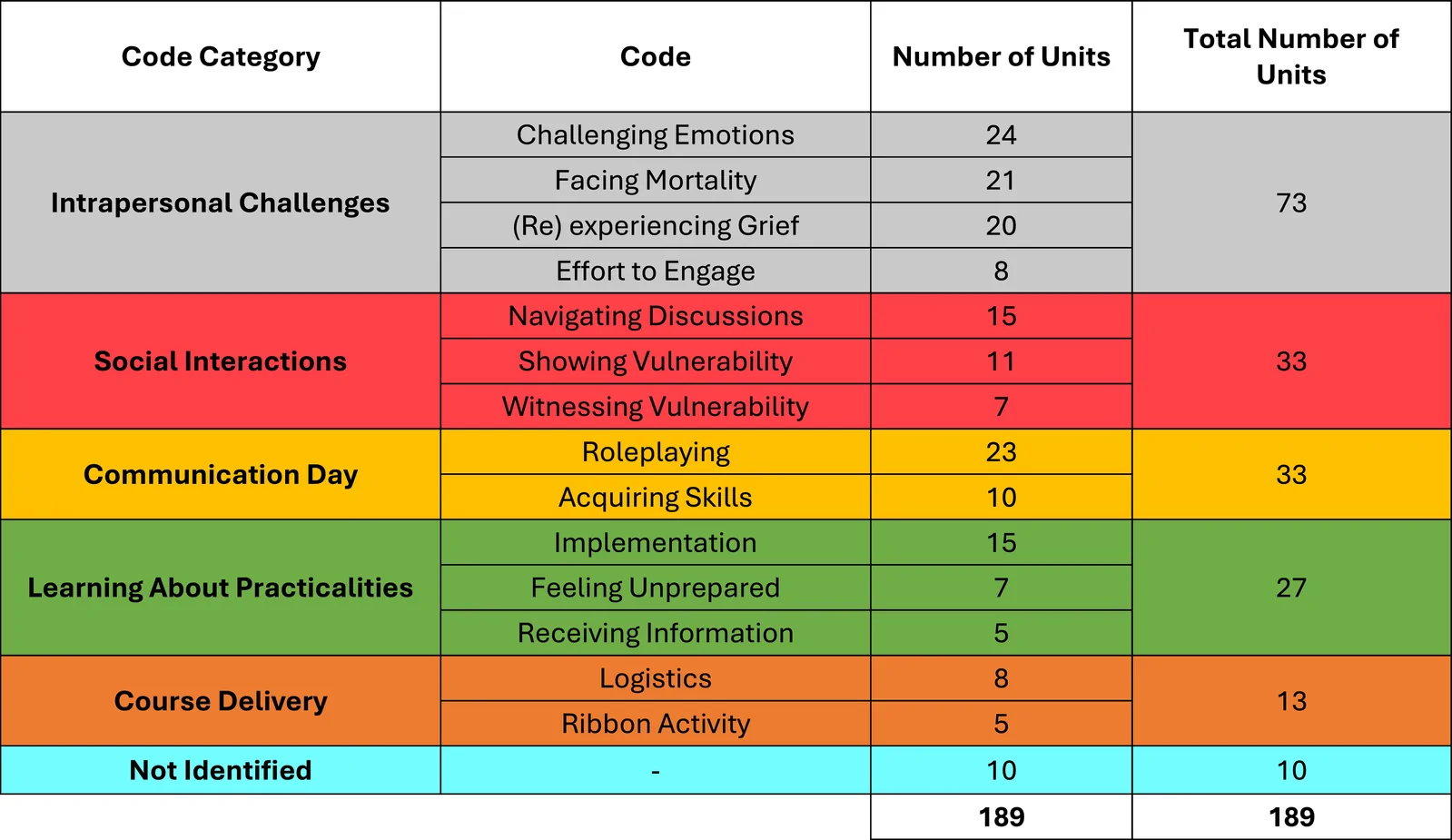
Table showing content analysis of responses to question 8 (what has been the most challenging part of the course?) as number of data units for each code category and code. (-) indicates a code was not required to further divide and describe the data. Each colour corresponds to the named code category.

**Figure 6.**
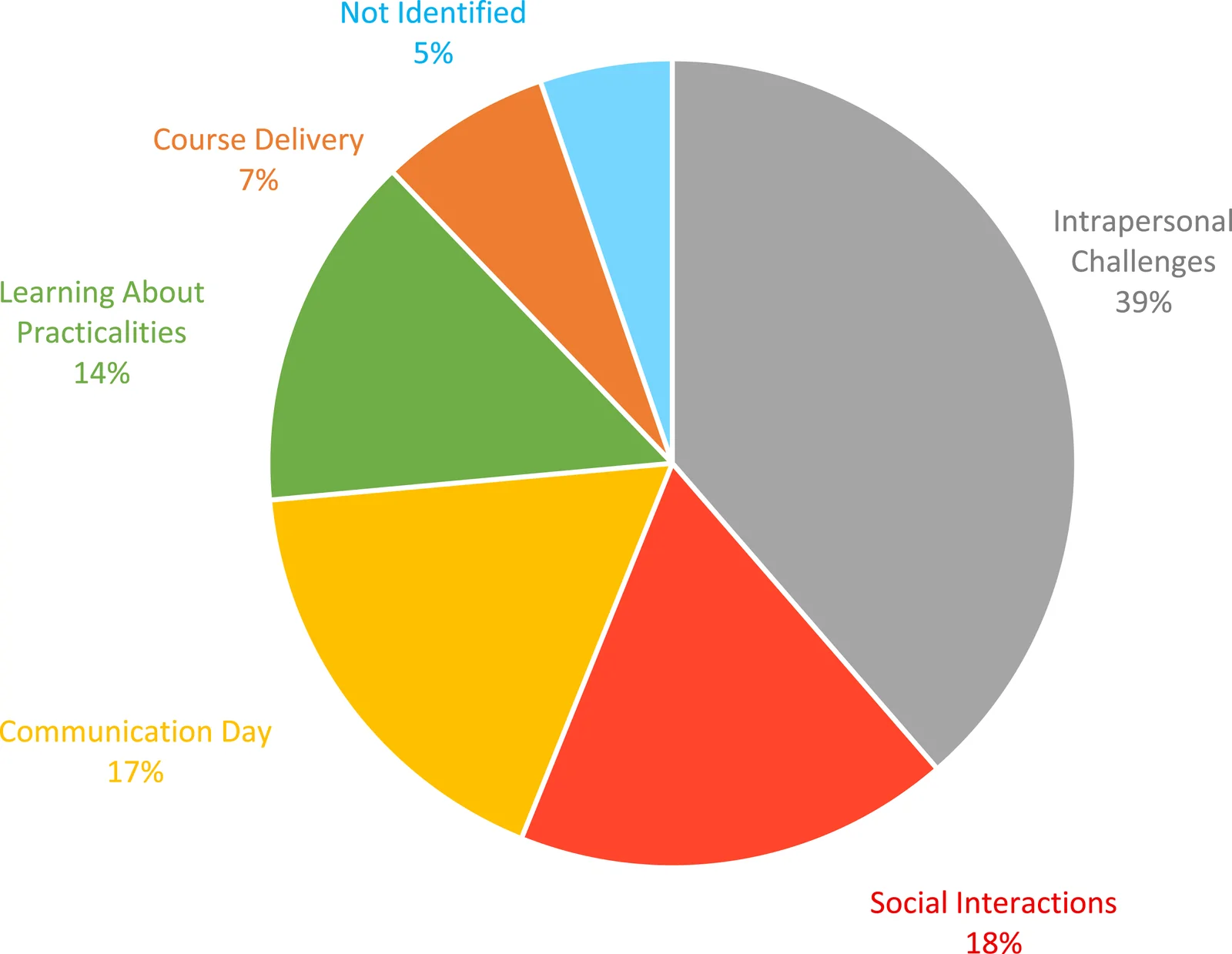
Pie chart showing content analysis of responses to question 8 (what has been the most challenging part of the course?) by proportion of data units (%) assigned to each code category. Each colour corresponds to the named code category.

The most prevalent code category was *Intrapersonal Challenges,* representing 73 units (39% of total). Sub-codes identified were *Challenging Emotions*, *Facing Mortality,* (*Re)experiencing Grief* and *Effort to Engage*:

“Thinking of myself and my own end of life” – Participant 65 *(Intrapersonal Challenges, Facing Mortality)*

“Facing my own historical grief” – Participant 32 *(Intrapersonal Challenges, (Re)experiencing Grief)*

The *Social Interaction*s (33 units, 17% of total) and *Communication Day* (33 units, 17% of total) categories appeared with equal frequency. *Social Interactions* were divided into *Navigating Discussions, Showing Vulnerability,* and *Witnessing Vulnerability. Communication Day* was again divided into *Roleplaying* and *Acquiring Skills.*

“Opening up about my distress” *– Participant 4 (Social Interactions, Showing Vulnerability)*

“Hearing others stories observing their grief” – *Participant 33 (Social Interactions, Witnessing Vulnerability)*

“The role play – powerful doesn’t mean easy!” *- Participant 131 (Communication Day, Roleplaying)*

The next most prevalent category was *Learning About Practicalities (*27 units, 14% of total). Responses highlighted *Implementation, Feeling Unprepared* and *Receiving Information*. Other categories were *Course Delivery* (13 units, 7% of total), and where the most challenging part was *Not identified (*10 units, 5% of total).

### Qualitative synthesis

#### Social contact

*Social Interactions* were highlighted as both the “best” and “most challenging” aspect of the course. *Building Relationships, Small Group Work, Listening*, and *Togetherness* were only coded as “best” parts, whereas *Discussions*, *Showing Vulnerability* and *Witnessing Vulnerability* were categorised as both “best” and “most challenging” parts. No aspects of *Social Interactions* were only coded as “most challenging”. Notably, *Social Interactions* were more commonly highlighted as a “best” part rather than a “most challenging” part.

Similarly, participants highlighted the *Communication Day* under both the “best” and “most challenging” aspect of the course in similar numbers, with *Roleplaying* dominating over *Acquiring Skills* in both “best” and “most challenging”. Another social aspect, course *Facilitation* (under *Course Delivery*), was only highlighted as a “best” part of the course.

#### Learning about death

*Learning About Practicalities* was slightly more commonly highlighted as the “most challenging” part of the course than the “best” part. Most of those highlighting it as a “best” part, found it prompted them to plan, whilst those indicating it was the “most challenging” part, found the information highlighted their unpreparedness and was intimidating to implement.

*Intrapersonal Challenges*, such as emotional responses and re-experiencing grief, was the most prevalent category highlighted as “most challenging”, whereas *Personal Growth*, which included emotional responses, was one of the least prevalent categories highlighted under “best” parts of the course. *Facing Mortality* (under *Intrapersonal Challenges*) was slightly more commonly identified as the “most challenging” part of the course than a “best” part (under *Death Dialogue*).

#### Attending a group course

*Course Delivery* was more often highlighted as the “best” part of the course than the “most challenging” part. Those highlight it as the “best” part focused on the course design and expertise of facilitators, whereas those highlighting it as the “most challenging” part focused on logistics e.g. timings.

In contrast, *The Whole Experience* and the *Atmospher*e were only coded as “best” parts of the course. Additionally, there was a proportion of responses indicating that they couldn’t identify a “most challenging” part of the course (*Not Identified)*, whereas inability to identify a “best” part of the course was not evident.

## Discussion

We found that The Omega Course increases self-reported comfort and preparedness around death and dying, suggesting it may aid in building the knowledge and skill components of death literacy. Therefore, **the first null hypothesis is rejected** (self-reported comfort and preparedness around dying will not significantly change pre-to post-course).

We also found that social connection and communication are elements central to compassionate community development^16^ suggesting that The Omega Course provides opportunity and impetus for compassionate community creation. Therefore, **the second null hypothesis is also rejected** (participants will not highlight social connections as key parts of their experience).

Our findings align with a large body of research indicating death educational interventions are feasible and positively received by the public.^18,20,21,23,24^ Our quantitative and qualitative findings also align with an abundance of research that death educational courses (such as Last Aid) promote attendee-reported comfort around death and dying,^18,21–24^ although these studies did not make use of pre *and* post course questionnaires to quantify such promotion.

Curiously, our qualitative findings contrast with the previous thematic analysis of Omega Course attendee feedback, which did not find social interaction and/or communication emerged as a key theme in course attendee qualitative feedback.^24^ This is perhaps because the qualitative questions posed in this previous study were more closed in nature, preventing themes of social connection and communication from being articulated. This previous study also made use of a retrospective design in which participants were invited to compete the feedback questionnaires up to 27 months after completing the course.^24^ It is therefore possible that social connection may be perceived as a more salient component of the course when data is collected immediately upon completion rather than months later. Finally, this previous study was limited to 23 participants,^24^ which may not have been large enough to allow themes of social interaction to emerge.

However, our qualitative findings *are* consistent with the two previous qualitative explorations of Last Aid participant experiences available. Both of these studies revealed social support was perceived by participants as important parts of the course experience.^22,23^ Specifically, Giehl et al. interviewed five female Last Aid course participants in Germany and Denmark. Analysis indicated “Support from Other Course Participants” which took the form of expressing empathy and sharing similar experiences, was reported as a key course experience by participants.^22^ Furthermore, Macaden et al. collected data from Last Aid participants and facilitators in the UK including survey feedback from 26 participants, and semi-structured interviews with an undisclosed number of participants. Their analysis indicated “Connectedness” to other participants and facilitators was perceived as key to the course experience, which was enhanced when the course cohort was more diverse, and when the course was conducted face-to-face.^23^ These themes align with several key codes emerging from our content analysis: *Discussions, Togetherness,* and both *Witnessing* and *Showing Vulnerability* were highlighted by Omega Course attendees as the best parts of their experience. This suggests social interactions may be a key, shared ingredient of success across multiple group death educational courses.

Our results indicated social connection and communication are viewed by participants as the best, yet simultaneously most challenging, parts of The Omega Course. This suggests the challenging nature of social connection during the course may be key to the experience and its success. It is perhaps by creating a socially supportive and connected environment that participants become particularly receptive to learning about death and dying. Therefore, we infer that confronting the topics of death and dying in a supportive and connected social environment allows for a) individuals in the group to successfully acquire death and dying-related knowledge, skills, and understanding, and b) the group as a whole to become more intimate, cohesive and interlinked, providing the basis for a compassionate community.

These interpretations align with the concept of ‘death sociability’ as proposed by Lessard et al.^36^; shared experiences and recognition around death and dying promotes the formation of social connections, and these connections allow communities to grow and become more compassionate. Lessard et al.^36^ further posit that death sociability thrives in safe, shared spaces which enable and encourage attendees to push past personal taboos around death, and to explore death within its social context. Whilst death sociability and compassionate growth may occur ‘naturally’ within a community following a crisis (e.g. an unexpected death, a new terminal diagnosis), these shared, safe spaces may act prophylactically to prepare communities before such a crisis hits. Our results suggest that The Omega Course may thus act as one of these proposed safe spaces in which death sociability is fostered and from which a prepared compassionate community can burgeon *before* a crisis occurs. Thus, The Omega Course may indeed be an effective tool for enabling communities to respond to the rising demand for palliative care.

## Limitations

This is the first study to quantify and statistically evaluate the impact of a death education intervention on participants’ death-related attitudes and integrate these with qualitative exploration among a larger sample. However, several limitations are considered below.

### Sample

The present findings are limited by lack of sample diversity as participants were predominantly older and female. This may reflect participant self-selection bias based on gendered attitudes about who is expected and/or needed to be death literate.^37^ This may also reflect participant self-selection bias based on age, as end-of-life planning is perceived as less relevant amongst younger cohorts.^7,38^ Furthermore, demographic data on ethnicity and socioeconomic status were not collected. All but two courses to date were held in affluent towns, suggesting findings may be limited to a wealthier cohort. This may limit generalisability of the present findings to poorer, younger and/or male and gender diverse cohorts, where crucially palliative resources already tend to be disproportionally scarcer.^4,39^ Unfortunately, such sample diversity limitations are mirrored amongst other death education course evaluations,^18^ highlighting a potential growing health inequality.

Participant profession was not collected, limiting generalisability to the general public. A previous large scale analysis of a death literacy intervention open to the general public (Last Aid) found a proportion of participants (9.4%) worked in the medical field.^18^ Given that participants experienced in healthcare are known to have a higher baseline death literacy,^12^ their experience of The Omega Course and scope for attitude change may be different to those approaching the course more naïve to its subject matter.

### Quantitative analysis and measurement tool

This study represents the first time this questionnaire has been used to evaluate pre- and post-course attitudes. Whilst appearing acceptable to participants in this study, the tool has not been validated and so may not accurately capture changes in death literacy. A valid, reliable and acceptable tool for capturing death literacy is now available: the Death Literacy Index (or its’ shortened form the Death Literacy Index-9).^12,13,40^ However, at the time the present study began, such a tool was not available and as such was not utilised.

With regard to compassionate community development, quantitative tools to capture the development of compassionate communities remain undefined.^41^

In addition, as previously described, 9.55% of qualitative data responses were missing for unknown reasons i.e. Missing Not at Random (MNAR).^34^ Declining to provide responses may reflect less engaged participants and/or those with less positive course experiences declining to complete the questionnaire(s),^42^ and thus skew the results more positively in favour of increased death literacy.

Finally, no a priori sample size calculation was undertaken, therefore the study was not prospectively powered to detect a predefined effect size.

### Qualitative analysis

As previously described, efforts were made to main trustworthiness in line with Lincoln and Guba’s criteria,^33^ minimising confirmation bias in the initial findings, and enhancing confirmability via the Stakeholder Engagement Meeting.^43^ However, as the content analysis did not involve direct clarification with participants, the initial content analysis findings could have been missing vital context.^32^ Furthermore, questions were not tailored towards exploration of compassionate communities, or mechanism of actions, and thus may have failed to capture this process in full. In addition, it is possible involvement of the course founder during the Stakeholder Engagement meeting could have introduced confirmation bias. To mitigate this, the lead and third authors were aware of this potential from the start of the study, designing the mixed-methods so that the explanatory value of the second author could be maximised while minimising the potential for favourable interpretations.”

### Longitudinal application

Post-course responses were collected immediately on completion of the last course session. Whilst this effectively limits recall bias, it limits understanding as to whether The Omega Course can engender medium or long-term changes in participants, or whether such changes translate into action e.g. talking to friends and family about wishes for death. Furthermore, collecting data immediately after the final session may more effectively reflect the participant’s experience of the final session rather than the course as a whole.

## Recommendations for research

Further evaluation of The Omega Course could make use of the Death Literacy Index to provide evidence as to whether the course builds death literacy,^12^ or if improvements are only seen in self-reported comfort and preparedness. If the length of such a tool is off-putting for use among the study population, a shortened, reliable, and validated 9-item version of the Death Literacy Index is also available^40^

Further evaluations could also employ in-depth and prolonged direct participant engagement to better understand and represent participant’ experiences. Such data could help clarify whether participants understand social connections as a mechanism by which they built knowledge and understanding, and perhaps *which* types or aspects of social connection are perceived as more or less useful in this process. Such in-depth contact could similarly be made with course facilitators, to help understand whether participants and facilitators have a shared understanding of how the course works, and whether facilitators can accurately perceive variation amongst participant experiences and consequently tailor their approach.

In addition, further evaluation of The Omega Course should make efforts to better capture participant demographics e.g. ethnicity, socioeconomic background, self-described gender, profession and/or prior experience with end-of-life services. This could help to better quantify which groups are drawn to the course, and whether some groups experience the course differently. Such data could also help to quantify, and then prevent, potential health inequalities in which some groups are less likely to seek out and receive end of life care education and preparation.

Finally, further research could make use of longitudinal qualitative and qualitative data to help determine whether the course’s potential impact on participants (and their understanding of this impact) changes over time, and whether participation in the course leads to actions e.g. expressing end-of-life preferences informally and/or legally.

## Implications for practice

Our findings suggest The Omega course builds comfort and preparedness around death and dying with small to moderate effect sizes. Therefore, we suggest the course is an acceptable and effective group intervention for use in community settings as a tool for death education and could be offered to more communities to help meet the growing need for death literacy.

Our interpretations suggest social connection and death sociability is key in the success of The Omega Course. Therefore, we suggest facilitators of death education courses should prioritise and promote social interaction during sessions. This could involve implementing further social icebreaker activities during all sessions, intentionally creating more opportunities for informal conversation before, during, and after sessions, and prioritising group discussions and interactions over prescribed content (e.g. worksheets, videos) where appropriate.

As previously discussed, course attendees appear to be predominately female, older and affluent, suggesting the course is at risk of widening a health inequality amongst more diverse populations.^4,39^ Therefore, we suggest death education courses should prioritise the recruitment of diverse cohorts. This could involve actively advertising to younger and more diverse communities e.g. via social media, universities and colleges, religious organisations, voluntary and third-sector organisations, and culturally specific community groups. Secular, inner-city venues, or further online courses could also be considered to enhance accessibility for these groups.

## Conclusion

This study highlighted the significant potential of The Omega Course to enhance death literacy and foster compassionate communities. Participants’ death-related comfort and preparedness increased post-course, with social connections identified as challenging, yet crucial, in achieving this. Thus, death sociability may be the mechanism by which the course promotes community growth. This study indicates structured educational interventions like The Omega Course could play a critical role in empowering individuals to engage with end-of-life issues, thus providing impetus for compassionate community development. This represents a step towards fostering a society prepared to support its members both in living and dying.

## Supplemental material

Supplemental material - Cultivating death literacy and compassionate communities via ‘The Omega course’: A mixed methods exploration Supplemental material for Cultivating death literacy and compassionate communities via ‘The Omega course’: A mixed methods exploration by Suzannah Laws, Chantal Meystre, John MacArtney in Palliative Care and Social Practice

Supplemental material - Cultivating death literacy and compassionate communities via ‘The Omega course’: A mixed methods exploration Supplemental material for Cultivating death literacy and compassionate communities via ‘The Omega course’: A mixed methods exploration by Suzannah Laws, Chantal Meystre, John MacArtney in Palliative Care and Social Practice

## Data Availability

All tools used are available in the supplementary materials. Data can be made available on request to the corresponding author.*
