# Supplementary material for: Cultivating death literacy and compassionate communities via ‘The Omega course’: A mixed methods exploration
Source: Palliat Care Soc Pract. 2026 Sep 17;20:26323524261481869. doi: 10.1177/26323524261481869 (PMC13724883; doi:10.1177/26323524261481869)
Supplement: Supplementary file 1 [file sj-pdf-1-pcr-10.1177_26323524261481869.pdf]

Supplementary Materials

Supplementary Material 1: Overview of Omega Course Syllabus

STRUCTURE, CONTENT, AND INTENT OF THE OMEGA COURSE			
**TOPIC**	**SET UP**	**CONTENT**	**AIM**
**Session 1**<br>**Learning to face the fact of death**<br>**2-3 hours**	Café style discussions interspersed with taught elements, films, time for discussion and plenary feedback.	Introductions and hopes and fears for the course in the round.<br>Quiz of recent celebrity deaths. Discussion of personal experiences and any sequelae from them. Videos of why it is good to plan for dying and its benefits. Discussion of film content.	To introduce the concept of universal mortality and debrief previous experiences of death and dying. To begin to develop comfort with the fact of universal mortality.
**Session 2**<br>**Living while dying**<br>**2-3 hours**	Café style discussions interspersed with taught elements, films, time for discussion and plenary feedback.	Check-in. Film on the need for honest answers to the dying. Common regrets of the dying followed by personal discussion.<br>Looking at life expectancy and one's place on that timeline. Discussing and planning bucket list aspirations.	Celebrating life to the full and reviewing its legacy, and planning what we want to do before dying.
**Session 3**<br>**Talking practicalities**<br>**2-3 hours**	Café style discussions interspersed with taught elements, films, time for discussion and plenary feedback.	Thinking about what sort of treatments one would want. Describing different types of advance care planning and their benefits and pitfalls. Explaining Advance Decision to Refuse Treatment, Lasting Powers of Attorney, and practical issues of Mental Capacity. Using the Omega Action Sheet to choose which elements of planning are personal priorities for setting affairs in order, including wills and digital legacy. Exploring an example of care for someone without full mental capacity.	Share information about Advance Care Planning and how to make it legally binding should one wish to do so.
**Session 4**<br>**How to talk to people who are sick or**	A whole group experience in the round followed by two-person role	Group sharing of what is found hard to handle socially when encountering the dying and bereaved. Teaching the principles of good communication with a	Teach good communication followed by experiential role play to enable confidence in talking about dying and bereavement using better

**bereaved**<br><br>**6.5 hours**	play with the remainder of the group observing and giving feedback.	focus on listening to the other's agenda, reflecting and clarifying with empathy. Then discussing the attendees own challenging communication scenarios to collectively plan role play. Responsibility for the content lies with the group not with the role player. This is kept safe by clear positive feedback rules and careful facilitation with 'timeout' options.	ways of communicating to ensure attendees are not lost for words in the face of a dying friend or bereaved person.
**Session 5**<br><br>**Supporting people who are dying or bereaved.**<br><br>**2-3 hours**	Café style discussions interspersed with taught elements, films, time for discussion and plenary feedback.	The course ends by looking at the pain of bereavement, grief theories, anticipatory grief, and what can be usefully done to help.<br><br>Postcards with personally recorded intentions are collected and sent back to participants 6 weeks later.<br><br>The course finale rounds up the course learning and assesses the course objectives. Attendees are invited to a social event.	To understand the needs of the bereaved and become more confident meeting them.<br><br>Making personal post-course intentions.
**Social event**	Informal buffet style meal with facilitators, previous attendees, and enquirers for the next course.	No formal agenda but a time to discuss and comment on the course during the evening and ask questions.	To further develop friendships and encourage resolve and outreach to be better family, friends and neighbours in the community.

Supplementary Material 2: Strengthening the Reporting of Observational Studies in Epidemiology (STROBE) Reporting Guidelines Completed Checklist

	Item No	Recommendation	Page No
**Title and abstract**	1	(*a*) Indicate the study's design with a commonly used term in the title or the abstract	1
		(*b*) Provide in the abstract an informative and balanced summary of what was done and what was found	1
**Introduction**			
Background/rationale	2	Explain the scientific background and rationale for the investigation being reported	1-2
Objectives	3	State specific objectives, including any prespecified hypotheses	2
**Methods**			
Study design	4	Present key elements of study design early in the paper	2-3
Setting	5	Describe the setting, locations, and relevant dates,	2-5

		including periods of recruitment, exposure, follow-up, and data collection	
Participants	6	(*a*) Give the eligibility criteria, and the sources and methods of selection of participants	3
Variables	7	Clearly define all outcomes, exposures, predictors, potential confounders, and effect modifiers. Give diagnostic criteria, if applicable	3-4
Data sources/ measurement	8*	For each variable of interest, give sources of data and details of methods of assessment (measurement). Describe comparability of assessment methods if there is more than one group	3-5
Bias	9	Describe any efforts to address potential sources of bias	5
Study size	10	Explain how the study size was arrived at	14
Quantitative variables	11	Explain how quantitative variables were handled in the analyses. If applicable, describe which groupings were chosen and why	2-5
Statistical methods	12	(*a*) Describe all statistical methods, including those used to control for confounding	4
		(*b*) Describe any methods used to examine subgroups and interactions	N/A
		(*c*) Explain how missing data were addressed	4 & 6
		(*d*) If applicable, describe analytical methods taking account of sampling strategy	N/A
		(*e*) Describe any sensitivity analyses	6 & 26
**Results**			
Participants	13*	(a) Report numbers of individuals at each stage of study—eg numbers potentially eligible, examined for eligibility, confirmed eligible, included in the study, completing follow-up, and analysed	5-9
		(b) Give reasons for non-participation at each stage	5-9
		(c) Consider use of a flow diagram	N/A
Descriptive data	14*	(a) Give characteristics of study participants (eg demographic, clinical, social) and information on exposures and potential confounders	5 & 14
		(b) Indicate number of participants with missing data for each variable of interest	4 & 6
Outcome data	15*	Report numbers of outcome events or summary measures	5-12
Main results	16	(*a*) Give unadjusted estimates and, if applicable, confounder-adjusted estimates and their precision (eg, 95% confidence interval). Make clear which confounders were adjusted for and why they were included	6-7
		(*b*) Report category boundaries when continuous variables were categorized	N/A
		(*c*) If relevant, consider translating estimates of relative risk into absolute risk for a meaningful time period	N/A
Other analyses	17	Report other analyses done—eg analyses of subgroups and interactions, and sensitivity analyses	6 & 26
**Discussion**			
Key results	18	Summarise key results with reference to study objectives	12
Limitations	19	Discuss limitations of the study, taking into account sources of potential bias or imprecision. Discuss both direction and magnitude of any potential bias	14-15
Interpretation	20	Give a cautious overall interpretation of results considering objectives, limitations, multiplicity of	12-16

		analyses, results from similar studies, and other relevant evidence	
Generalisability	21	Discuss the generalisability (external validity) of the study results	12-16
**Other information**			
Funding	22	Give the source of funding and the role of the funders for the present study and, if applicable, for the original study on which the present article is based	7 & 16

*Give information separately for exposed and unexposed groups.

Supplementary Material 3: Standards for Reporting Qualitative Research (SRQR) Reporting Guidelines Completed Checklist

Title and abstract

	**Page/line no(s).**
**Title** - Concise description of the nature and topic of the study Identifying the study as qualitative or indicating the approach (e.g., ethnography, grounded theory) or data collection methods (e.g., interview, focus group) is recommended	1
**Abstract** - Summary of key elements of the study using the abstract format of the intended publication; typically includes background, purpose, methods, results, and conclusions	1

Introduction

**Problem formulation** - Description and significance of the problem/phenomenon studied; review of relevant theory and empirical work; problem statement	1-2
**Purpose or research question** - Purpose of the study and specific objectives or questions	1-2

Methods

**Qualitative approach and research paradigm** - Qualitative approach (e.g., ethnography, grounded theory, case study, phenomenology, narrative research) and guiding theory if appropriate; identifying the research paradigm (e.g., postpositivist, constructivist/ interpretivist) is also recommended; rationale**	2-3
**Researcher characteristics and reflexivity** - Researchers' characteristics that may influence the research, including personal attributes, qualifications/experience, relationship with participants, assumptions, and/or presuppositions; potential or actual interaction between researchers' characteristics and the research questions, approach, methods, results, and/or transferability	5 & 14-15
**Context** - Setting/site and salient contextual factors; rationale**	1-5
**Sampling strategy** - How and why research participants, documents, or events were selected; criteria for deciding when no further sampling was necessary (e.g., sampling saturation); rationale**	3

**Ethical issues pertaining to human subjects** - Documentation of approval by an appropriate ethics review board and participant consent, or explanation for lack thereof; other confidentiality and data security issues	5-6 & 16
**Data collection methods** - Types of data collected; details of data collection procedures including (as appropriate) start and stop dates of data collection and analysis, iterative process, triangulation of sources/methods, and modification of procedures in response to evolving study findings; rationale**	3
**Data collection instruments and technologies** - Description of instruments (e.g., interview guides, questionnaires) and devices (e.g., audio recorders) used for data collection; if/how the instrument(s) changed over the course of the study	3 & 25
**Units of study** - Number and relevant characteristics of participants, documents, or events included in the study; level of participation (could be reported in results)	6
**Data processing** - Methods for processing data prior to and during analysis, including transcription, data entry, data management and security, verification of data integrity, data coding, and anonymization/de-identification of excerpts	3-5
**Data analysis** - Process by which inferences, themes, etc., were identified and developed, including the researchers involved in data analysis; usually references a specific paradigm or approach; rationale**	2-5
**Techniques to enhance trustworthiness** - Techniques to enhance trustworthiness and credibility of data analysis (e.g., member checking, audit trail, triangulation); rationale**	5

Results/findings

**Synthesis and interpretation** - Main findings (e.g., interpretations, inferences, and themes); might include development of a theory or model, or integration with prior research or theory	12-16
**Links to empirical data** - Evidence (e.g., quotes, field notes, text excerpts, photographs) to substantiate analytic findings	11-1

Discussion

**Integration with prior work, implications, transferability, and contribution(s) to the field -** Short summary of main findings; explanation of how findings and conclusions connect to, support, elaborate on, or challenge conclusions of earlier scholarship; discussion of scope of application/generalizability; identification of unique contribution(s) to scholarship in a discipline or field	13-16
**Limitations** - Trustworthiness and limitations of findings	14-15

Other

**Conflicts of interest** - Potential sources of influence or perceived influence on study conduct and conclusions; how these were managed	5 & 14-16
**Funding** - Sources of funding and other support; role of funders in data collection, interpretation, and reporting	7 & 16

Supplementary Material 4: Good reporting of a mixed-methods study (GRAMMS) checklist

Guideline	Section: page(s)
Describe the justification for using a mixed methods approach to the research question	Introduction: 1-2
Describe the design in terms of the purpose, priority and sequence of methods	Methods: 2-5
Describe each method in terms of sampling, data collection and analysis	Methods: 2-5
Describe where integration has occurred, how it has occurred and who has participated in it	Methods: 2-5<br>Results: 14<br>Discussion: 12
Describe any limitation of one method associated with the present of the other method	Discussion: 14-15
Describe any insights gained from mixing or integrating methods	Results: 14<br>Discussion: 14-15

Supplementary Material 5: Participant Pre- and Post-Course Questionnaires

Pre-course questionnaire:

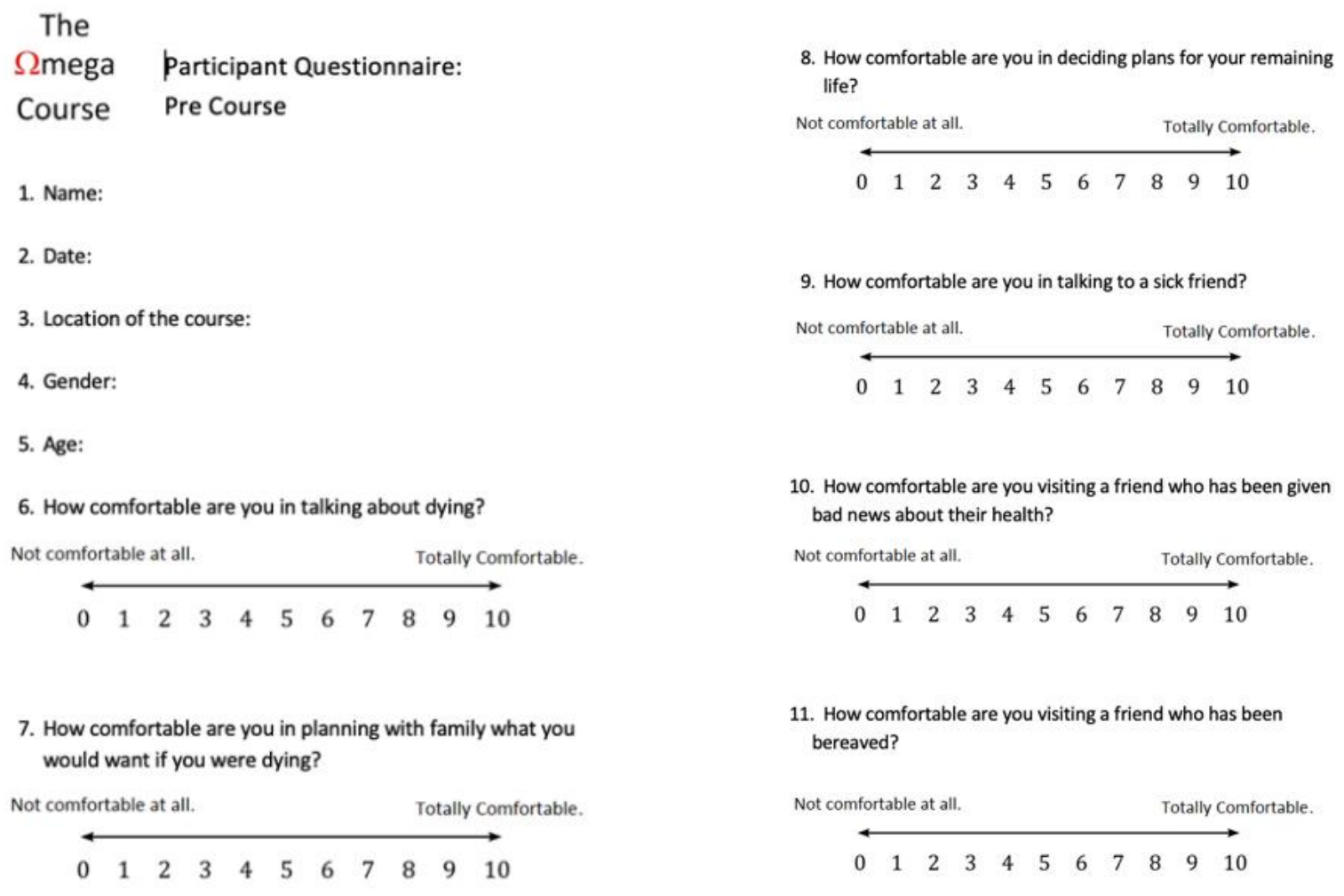
The Ωmega Course

Participant Questionnaire:
Pre Course

1. Name:
2. Date:
3. Location of the course:
4. Gender:
5. Age:
6. How comfortable are you in talking about dying?

Not comfortable at all. ←→ Totally Comfortable.
0 1 2 3 4 5 6 7 8 9 10

7. How comfortable are you in planning with family what you would want if you were dying?

Not comfortable at all. ←→ Totally Comfortable.
0 1 2 3 4 5 6 7 8 9 10

8. How comfortable are you in deciding plans for your remaining life?

Not comfortable at all. ←→ Totally Comfortable.
0 1 2 3 4 5 6 7 8 9 10

9. How comfortable are you in talking to a sick friend?

Not comfortable at all. ←→ Totally Comfortable.
0 1 2 3 4 5 6 7 8 9 10

10. How comfortable are you visiting a friend who has been given bad news about their health?

Not comfortable at all. ←→ Totally Comfortable.
0 1 2 3 4 5 6 7 8 9 10

11. How comfortable are you visiting a friend who has been bereaved?

Not comfortable at all. ←→ Totally Comfortable.
0 1 2 3 4 5 6 7 8 9 10

Post-course questionnaire:

Omega Participant post-course questionnaire

1. Name:
2. Date:
3. Location:
4. Gender:
5. Age:
6. How comfortable are you in talking about dying?

Not comfortable at all. — Totally Comfortable.

0 1 2 3 4 5 6 7 8 9 10

7. How comfortable are you in planning with family what you would want if you were dying?

Not comfortable at all. — Totally Comfortable.

0 1 2 3 4 5 6 7 8 9 10

We take your privacy very seriously. Please see our displayed privacy notice. We will use your data only to contact you with details of courses, events and occasional evaluations which may be used for research.

8. How comfortable are you in deciding plans for your remaining life?

Not comfortable at all. — Totally Comfortable.

0 1 2 3 4 5 6 7 8 9 10

9. How comfortable are you in talking to a sick friend?

Not comfortable at all. — Totally Comfortable.

0 1 2 3 4 5 6 7 8 9 10

10. How comfortable are you visiting a friend who has been given bad news about their health?

Not comfortable at all. — Totally Comfortable.

0 1 2 3 4 5 6 7 8 9 10

11. How comfortable are you visiting a friend who has been bereaved?

Not comfortable at all. — Totally Comfortable.

0 1 2 3 4 5 6 7 8 9 10

We take your privacy very seriously. Please see our displayed privacy notice. We will use your data only to contact you with details of courses, events and occasional evaluations which may be used for research.

12. What has been the best part of the course?

13. What has been the most challenging part of the course?

14. Are there any comments you would feel happy for us to share with others thinking of coming on the course in the future?

15. Was the venue comfortable?

Not comfortable at all. — Totally Comfortable.

0 1 2 3 4 5 6 7 8 9 10

We take your privacy very seriously. Please see our displayed privacy notice. We will use your data only to contact you with details of courses, events and occasional evaluations which may be used for research.

Supplementary Material 6: Parametric test assumptions

Data provided for Questions 1-6 violated the Shapiro-Wilk test of normality (all ps <0.001). However, Field (42) argues that tests for normality in samples of this size are often significant due to minor and meaningless deviations from normality. Instead, Field suggests that normality assumptions for such sample sizes are unnecessary and instead the central limit theorem can be relied upon. In addition, Q-Q plots (see below) appeared normal. Therefore, parametric t-tests were used.

Quantile-Quantile (Q-Q) Plots for Q1-6:

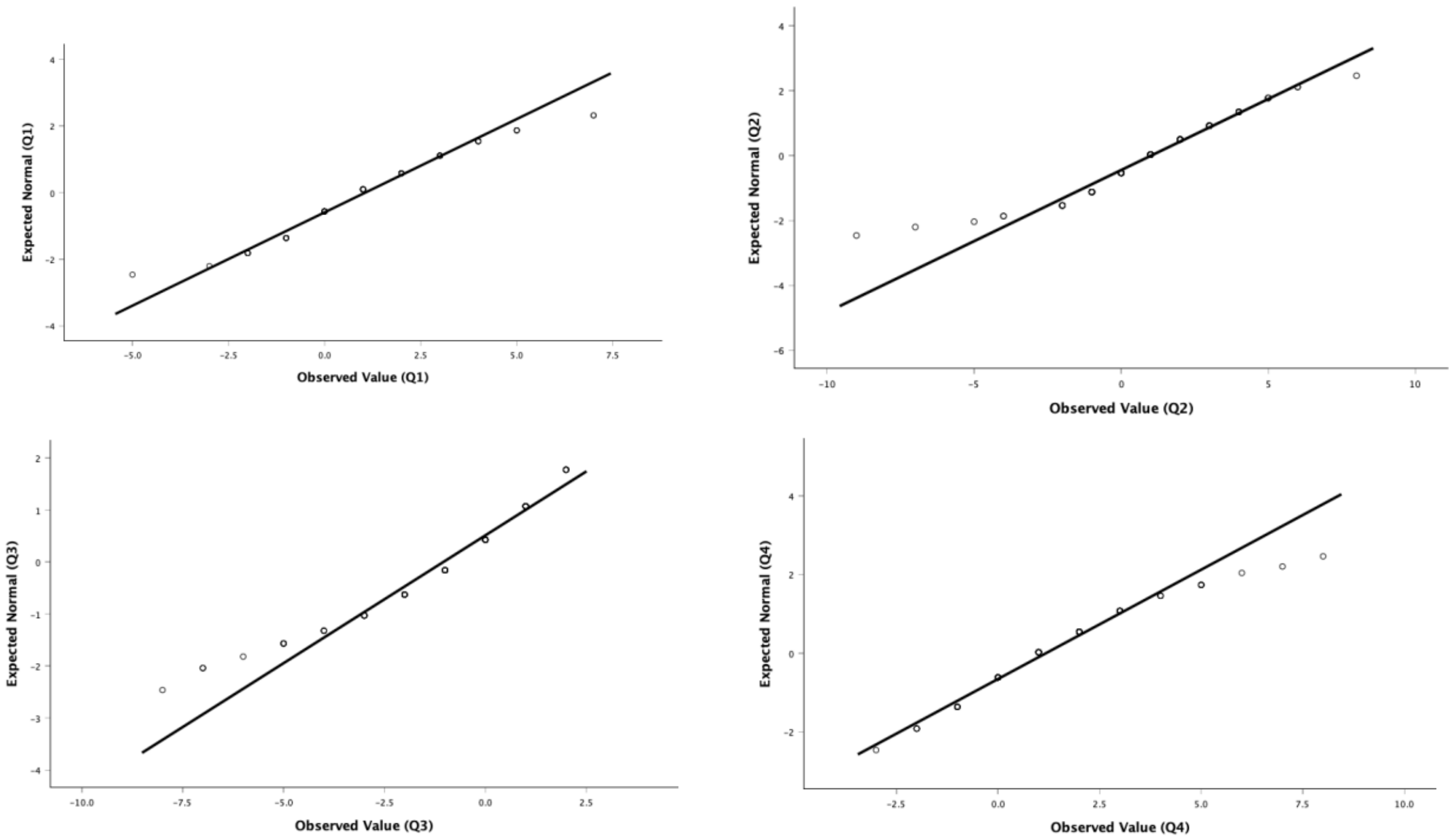

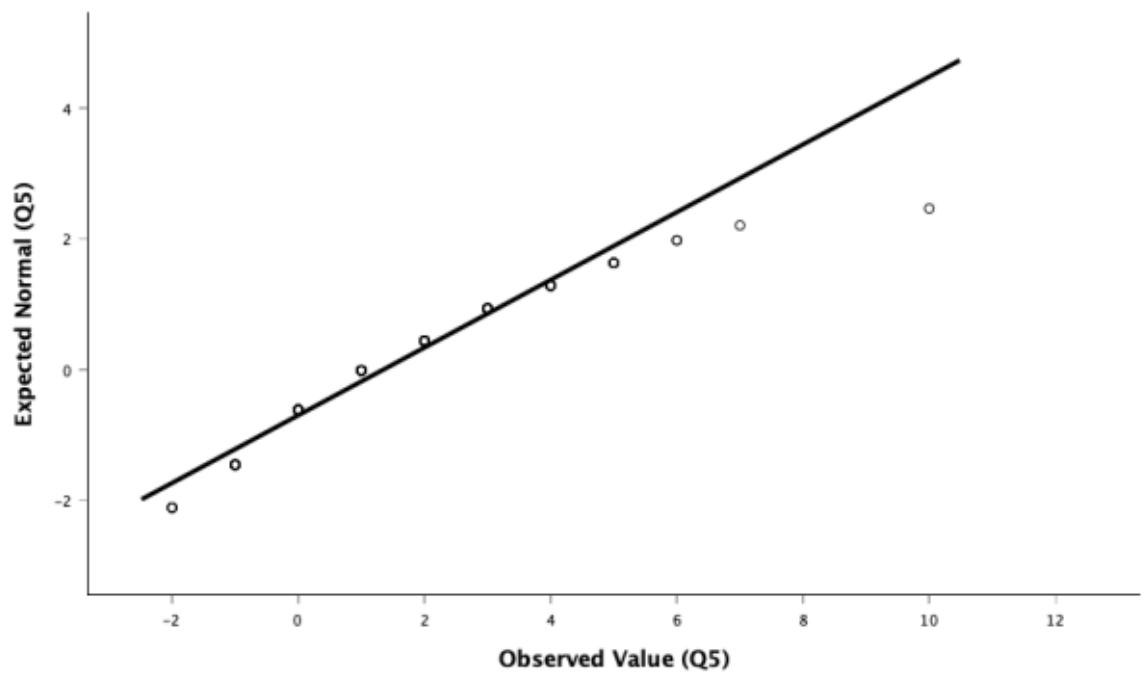

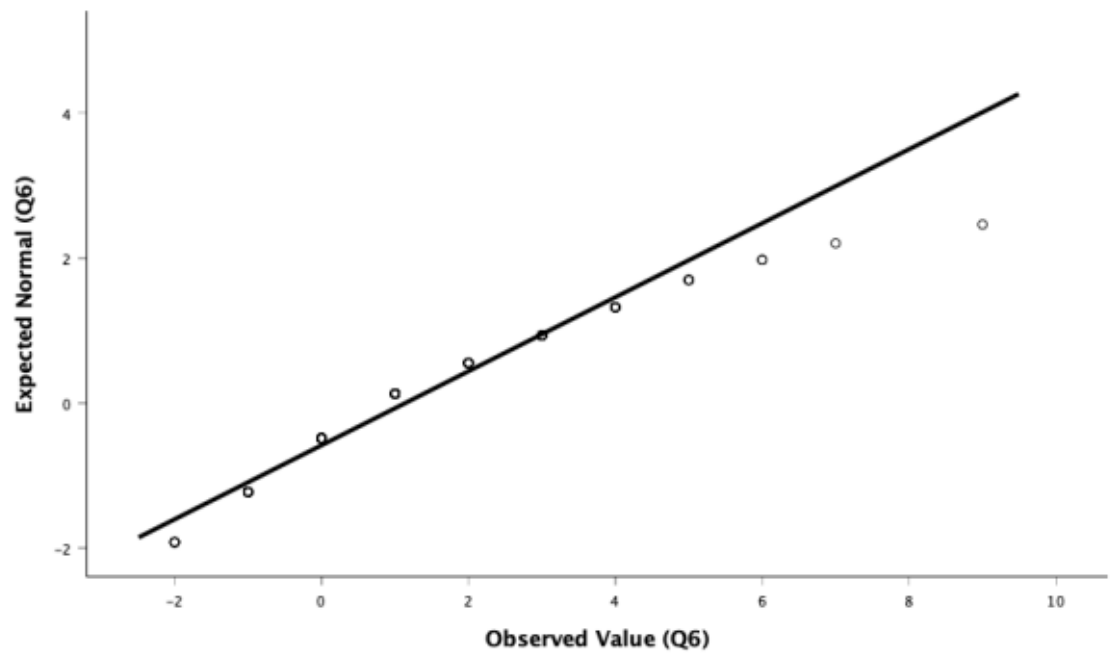

In the interests of transparency and robustness, pre- to post-course score comparisons were repeated using the equivalent non-parametric test, the Wilcoxon Signed Rank test. Wilcoxon Signed Rank analysis revealed participant scores post course were significantly higher than the pre course scores for all questions: Q1 (Z=6.43, p<0.001), Q2 (Z=5.56, p<0.001), Q3 (Z=5.72, p<0.001), Q4 (Z=6.85, p<0.001), Q5 (Z=7.30, p<0.001), Q6 (Z=6.18, p<0.001).

Supplementary Material 7: Code book for Question 6 (What has been the best part of the course?)

Code Category	Code	Sub Code	Definition	Examples
Social Interactions	Discussions		Engaging in discussion(s) with other participants during the course	“Discussions on many topics” – Participant 114 “The conversations with others.” – Participant 131
	Building Relationships		Forming connections with other participants during the course	“Meeting interesting people.” – Participant 103 “nice to meet and get to know a really lovely group of people” – Participant 121
	Togetherness		A sense of belonging or shared experience within the group	“being part of a group” – Participant 111 “a real sense of togetherness” - Participant 10
	Listening		Hearing others’ perspectives, experiences, or ideas	“Hearing others experiences and journeys” – Participant 111 “Hearing others points of view.” – Participant 124
	Witnessing Vulnerability		Observing the openness and honesty of other participants e.g. as they share personal stories or emotions	“Openness of contributors.” – Participant 105 “the frankness of individuals participating.” - Participant 61
	Showing Vulnerability		Experience when	“Sharing my story and

			offering one's own personal experiences, stories, or insights with the rest of the group	thoughts" – Participant 13<br>"Being able to talk about personal experience of grief" – Participant 109
	Small Group Work		Any activity in a small group	"Some frank sharing in the smaller groups." – Participant 46<br>"I enjoyed the breakout groups" – Participant 150
Course Delivery	Design		The structure and style of the course, or reference to specific content and activities with the course	"All lead perfectly from one to another" – Participant 97<br>"It has been an excellent mix of practical, emotional, theoretical, role play etc." – Participant 121
	Facilitators	Expertise	Any of the facilitators' knowledge, professional experience, and ability to provide valuable insights	"Valuable input from [Facilitator Name]'s [Facilitator Job Role] experience." – Participant 52<br>"The pearls of wisdom from [Facilitator Name] – the real-life stories to illustrate the theory." – Participant 135
		Leadership	Any of the facilitators' ability to effectively present, manage and organise the course	"Especially valued its organisation" – Participant 29<br>"presenters excellent" – Participant 95
		Support	Emotional encouragement, comfort, and/or support provided by any of the facilitators	"Support and consideration from the people running the course" – Participant 55<br>"Feeling so at ease with the facilitators" – Participant 47
Learning About Practicalities	Prompting to Plan	For Self	Causing participants to consider and/or make plans for their own future life and/or death	"it really made me think about making the most of my life from now on!" – Participant 1<br>"Planning my death and future life" – Participant 81
		For Friends & Family	Causing participants to consider and/or make plans for the future life and/or death of friends and/or family members	"Making me think about how to make my children's life easier by being organised" – Participant 116<br>"Being challenged to think how I can best spend time with a terminal ill friend" –

			Participant 51
	Receiving Information	Receiving knowledge or insights about the legalities and/or practicalities of death and dying	"Information about preparing/planning/variety of methods." – Participant 117<br>"Practical insights on legal aspects." – Participant 133
Communication Day	Roleplaying	Experiences of role-playing activities	"The role play – v. powerful!" – Participant 135<br>"Observing role play" – Participant 31
	Acquiring Skills	Learning specific techniques or general approaches to effective communication	"Learning about options/ways to approach difficult situations with people" – Participant 20<br>"I enjoyed the chats of how and what to say" – Participant 104
Death Dialogue	Finding Comfort	Becoming more at ease discussing death and dying	"Discussing with others the issues of death, and not feeling uncomfortable" – Participant 99<br>"I feel more confident if I'm ever in that position." - Participant 104
	Exploring	The opportunity to talk about death and dying	"Talking about taboo subjects" – Participant 63<br>"Conversing about death and dying" – Participant 111
The Whole Experience		The overall impact and benefit of the course, encompassing all aspects.	"All sessions have been positive and helpful." – Participant 10<br>"I have found all parts useful." – Participant 117
Personal Growth	Opportunity to Learn	Gaining of new knowledge, skills, or insights	"I feel I have been enabled to learn" – Participant 54<br>"Learning so much even though I have been involved with death and dying with my wok over last 15 years!" – Participant 108
	Emotional Challenges	Experiencing emotional responses to the course content	"Some emotional moments" – Participant 59<br>"Difficulty between professional and personal" – Participant 101

	Exceeding Expectations	Course surpassed participants' initial hopes or assumptions	"Better than I could have imagined." – Participant 45 "Much more comprehensive (and enjoyable) than I expected" – Participant 121
Safe Atmosphere		A supportive and welcoming environment where participants feel secure in expressing themselves	"Being made to feel welcome" – Participant 103 "In a friendly and supportive environment" – Participant 109

Appendix 8: Code book for Question 7 (What has been the most challenging part of the course?)

Code Category	Code	Definition	Examples
Intrapersonal Challenges	Challenging Emotions	Experiencing emotional responses to the course content	"Brought back emotions buried" – Participant 8 "My emotions." – Participant 56
	Facing Mortality	Confronting personal thoughts or fears about one's own death, ageing, or end-of-life	"Thinking of myself and my own end of life" – Participant 65 "Coming to terms with ageing" – Participant 63
	(Re)experiencing Grief	Revisiting past grief or loss experiences during the course	"Facing my own historical grief" – Participant 32 "Delving into my memories of when my father died" – Participant 8
	Effort to Engage	The mental and/or emotional effort required to actively participate in the course or overcome initial hesitations	"Intensive concentration required to stay focussed on the contents." – Participant 126 "Walking through the door on the 1st day." – Participant 86
Social Interactions	Navigating Discussions	Managing one's own behaviour when engaging in group discussions, including when/how to speak and/or listen	"Speaking out when going round the room" – Participant 68 "Listening patiently" – Participant 7

	Showing Vulnerability	Experience when offering one's own personal experiences, stories, or insights with the rest of the group	"Opening up about my distress" – Participant 4 "Fear of crying" – Participant 45
	Witnssing Vulnerability	Observing the openness and honesty of other participants e.g. as they share personal stories or emotions	"Hearing others stories observing their grief" – Participant 33 "listening to the experiences of others was at times challenging" – Participant 18
Communication Day	Roleplaying	Experiences of role-playing activities	"The role play – powerful doesn't mean easy!" - Participant 131 "The possibility of actually doing role play" – Participant 21
	Acquiring Skills	Learning specific techniques or general approaches to effective communication	"Made me think about my attitude and how what I say may affect someone" – Participant 111 "looking at how to react/talk/listen to people who have been bereaved/had bad news" – Participant 122
Course Delivery	Logistics	Practical aspects of attending the course, not concerned with course content or structure itself	"Getting there on time!" – Participant 47 "The noise and heat" – Participant 44
	Ribbon Activity	Experience of the ribbon task (activity in which participants reflect on how many more years they have until reaching average life expectancy using a length of ribbon)	"Anything referencing the past i.e. the ribbon exercise" – Participant 121 "Considering past experiences (ribbon exercise) and how this influences the future" – Participant 73
Learning About Practicalities	Implementation	Applying course learnings about practicalities of death to real-life situations and conversations after/outside of the course	"Going home and discussing it with my husband/family" – Participant 12 "I need to start sorting out some practical things with family" – Participant 62
	Feeling Unprepared	Realizing personal gaps in planning and/or preparedness	"Trying not to panic when I realise how much

		for end-of-life matters	I need to do" – Participant 40<br>"Confronting my lack of preparation re death" – Participant 109
	Receiving Information	Learning or revisiting information about practicalities of death	"Practical things that need talking about" – Participant 96<br>"Lasting Power of Attorney (due to lack of knowledge" – Participant 94
Not Identified		Does not report any challenges or difficulties during the course, or unable to identify the most challenging aspect	"Nothing difficult" – Participant 76<br>"None" – Participant 98
