# Supplementary material for: Cultivating death literacy and compassionate communities via ‘The Omega course’: A mixed methods exploration
Source: Palliat Care Soc Pract. 2026 Sep 17;20:26323524261481869. doi: 10.1177/26323524261481869 (PMC13724883; doi:10.1177/26323524261481869)
Supplement: Supplementary file 2 [file sj-pdf-2-pcr-10.1177_26323524261481869.pdf]

Omega

Participant post-course questionnaire

1. Name:

2. Date:

3. Location:

4. Gender:

5. Age:

6. How comfortable are you in talking about dying?

Not comfortable at all. Totally Comfortable.

0 1 2 3 4 5 6 7 8 9 10

7. How comfortable are you in planning with family what you would want if you were dying?

Not comfortable at all. Totally Comfortable.

0 1 2 3 4 5 6 7 8 9 10

We take your privacy very seriously. Please see our displayed privacy notice. We will use your data only to contact you with details of courses, events and occasional evaluations which may be used for research.

8. How comfortable are you in deciding plans for your remaining life?

Not comfortable at all. Totally Comfortable.

0 1 2 3 4 5 6 7 8 9 10

9. How comfortable are you in talking to a sick friend?

Not comfortable at all. Totally Comfortable.

0 1 2 3 4 5 6 7 8 9 10

10. How comfortable are you visiting a friend who has been given bad news about their health?

Not comfortable at all. Totally Comfortable.

0 1 2 3 4 5 6 7 8 9 10

11. How comfortable are you visiting a friend who has been bereaved?

Not comfortable at all. Totally Comfortable.

0 1 2 3 4 5 6 7 8 9 10

We take your privacy very seriously. Please see our displayed privacy notice. We will use your data only to contact you with details of courses, events and occasional evaluations which may be used for research.

12. What has been the best part of the course?

13. What has been the most challenging part of the course?

14. Are there any comments you would feel happy for us to share with others thinking of coming on the course in the future?

15. Was the venue comfortable?

Not comfortable at all. Totally Comfortable.

0 1 2 3 4 5 6 7 8 9 10

We take your privacy very seriously. Please see our displayed privacy notice. We will use your data only to contact you with details of courses, events and occasional evaluations which may be used for research.

The Ωmega Course

Participant Questionnaire: Pre Course

1. Name:

2. Date:

3. Location of the course:

4. Gender:

5. Age:

6. How comfortable are you in talking about dying?

Not comfortable at all. Totally Comfortable.

0 1 2 3 4 5 6 7 8 9 10

7. How comfortable are you in planning with family what you would want if you were dying?

Not comfortable at all. Totally Comfortable.

0 1 2 3 4 5 6 7 8 9 10

8. How comfortable are you in deciding plans for your remaining life?

Not comfortable at all. Totally Comfortable.

0 1 2 3 4 5 6 7 8 9 10

9. How comfortable are you in talking to a sick friend?

Not comfortable at all. Totally Comfortable.

0 1 2 3 4 5 6 7 8 9 10

10. How comfortable are you visiting a friend who has been given bad news about their health?

Not comfortable at all. Totally Comfortable.

0 1 2 3 4 5 6 7 8 9 10

11. How comfortable are you visiting a friend who has been bereaved?

Not comfortable at all. Totally Comfortable.

0 1 2 3 4 5 6 7 8 9 10
